# Exergy-economic assessment of a hybrid power, cooling and heating generation system based on SOFC

**DOI:** 10.1016/j.heliyon.2023.e16164

**Published:** 2023-05-19

**Authors:** Rahim Zahedi, Mohammad Mahdi Forootan, Rouhollah Ahmadi, Mansour Keshavarzzadeh

**Affiliations:** a Department of Renewable Energy and Environmental, University of Tehran, Tehran, Iran; b Department of Energy Systems Engineering, Iran University of Science and Technology, Tehran, Iran; c Faculty of Mechanical Engineering, Iran University of Science and Technology, Tehran, Iran

**Keywords:** Hybrid system, SOFC, Exergy, Exergoeconomic, Parametric analysis

## Abstract

In this research, a combined cycle using a solid oxide fuel cell system, a single-stage H_2_O–NH_3_ absorption chiller and a residential hot water HX, is developed for the electricity production, hot water and cooling all at the same time, and it is studied from an exergy, energy, and exergoeconomic standpoint. Performance of system under the design condition is analyzed and the mathematical model is simulated. After analyzing the results in the initial input mode, changing the fuel cell current density effect and fuel utilization factor on the system efficiency is evaluated. The result indicates that total energy is 4.418 kW, the total exergy efficiency is 37.8%. And the overall irreversibility is 1.650 kW. On the other hand, the air HX, fuel cell and water HX are designed as elements that must be given more attention than others from the exergoeconomic perspective, because they have nearly the most amount of price compared to other parts.

## Nomenclature

SOFC: Solid oxide fuel cell
CHP: Combined heating and power system
MCDM: Multi-criteria decision-making
ORC: Organic Rankine cycle
S–CO_2_: Supercritical carbon dioxide
HX: Heat exchanger
CCHP: Combined cooling-heating and power system
LTHS: Low-temperature heat source
CRF: Capital recovery factor
ARC: absorption refrigeration cycle

## 1 Introduction

Nowadays, coal and oil resources are increasingly scarce in the world, the search for renewable and pollution-free clean energy has become a key element goal in the construction and planning of all countries which is reflected in the REPowerEU, Plan of the European Union and the Inflation Reduction Act of the United States [1]. The first truly global energy crisis during the Russia-Ukraine conflict sparks an unprecedented surge in renewable energy, which leads to the expansion of renewable energy power in Europe expected to double in the next five years. According to the Renewables 2022 issued by the International Energy Agency. by early 2025, renewable energy will overtake coal as the world's largest source of electricity generation, while global fuel cell power generation capacity will also double [2]. Among these renewable energies, fuel cells have been proposed as a relatively new technology in energy conversion and among the types of fuel cells, solid oxide fuel cells (SOFC)s due to high operating temperature, high efficiency, and having waste heat with high-temperature potential to increase the efficiency of energy conversion systems, can be combined with gas turbines or other types of power generation cycles, such as low temperatures power, heat, and cold generation cycles, which in various studies combined cycle of solid oxide fuel cell and gas turbine [[3], [4], [5]], fuel cell and organic steam Rankin and Kalina cycle [[6], [7], [8]], fuel cell and H_2_O–NH_3_ absorption refrigerant cycles [[9], [10], [11]], and fuel cell and absorption cold production cycle [12,13] are investigated. At present, the "wind - hydrogen strategy" in Europe enjoys a sound application prospect. After analyzing data on green financing, COVID-19, and energy production, it was observed that the implementation of green financing in the fuel cell industry led to a 19% increase fuel cell energy production during the pandemic [14].

Resource Evaluation should be ahead of Resource development. The long-term trend of energy also plays a key role in assessment and development and site construction in the region. Due to the difficulty of energy transmission in the region, the energy demand of the Antarctic research station and shipping route has become a major problem [15]. The energy module which was put into use at the Taishan Station in 2019 is China's first domestic polar unmanned energy system which is put into operation. This system needs to burn 5 tons of jet fuel for a year to ensure the power supply of the research station. Therefore, the gas emission generated behind it and environmental pollution have brought new challenges to the ecology of Antarctica. The excessive emission of greenhouse gases causes the rising temperature, and then affects the overall emissions of greenhouse gases in Antarctica, resulting in a vicious cycle. In response to the newly proposed goal of "Carbon peak" and "Carbon neutrality", the construction of "zero carbon emission" research station in the different regions can better reflect many countries' attention to clean energy [16].

Green roofs have been extensively used as a passive strategy to reduce building energy consumption for heating and cooling. Green roofs are a type of roof system that incorporate vegetation, substrate, and drainage layers on top of a traditional roof structure [17]. The vegetation layer of a green roof reduces the solar energy transmitted into the building through a number of mechanisms. Leaves have higher solar reflection than the substrate, thus increasing the heat loss by higher emissivity rates. They also reduce the substrate's temperature by foliage shading, and can provide additional cooling through plant transpiration. As such, plants can significantly contribute to the net solar balance between a green roof and the building. Research has extensively demonstrated that vegetated green roofs are more effective than conventional roofs in reducing the heat transferred indoors, sometimes even more than building insulation [18].

The use of combined heat and power systems (CHP) or combined cooling heating and power systems (CCHP) due to the simultaneous need for electricity and heat or electricity and cooling, in buildings and industry, has received a lot of attention and because of the advantages of these systems in reducing energy consumption and costs, it is one of the best solutions that have good economic efficiency [19]. The efficiency of conventional power generation cycles is about 30% or less, and most of the stimulant energy is wasted in these systems [20], whereas it is possible to achieve better efficiencies by using CHP or CCHP, this efficiency reaches 75–80% in triple production systems [21]. In cogeneration systems, the waste energy of the power generator is mainly used to generate heating or cooling. For example, heat dissipation in the Rankine cycle condenser can be used as a stimulus for a heating generation subsystem or a cooling generation subsystem [22]. The most common type of heating generation subsystem is hot water HX and the most common thermal energy cooling subsystem is the absorption chiller or ejector-cooling cycle.

Among the research conducted in the field of CHP and CCHP, Adebayo et al. [23] have simulated a micro-scale cogeneration system with simultaneous stimulation of geothermal and solar energy from the energy, exergy, and exergoeconomic standpoint. Parikhani et al. [24] have studied the CCHP system with solar energy stimulant along with storage tank for two seasons of winter and summer from the energy, exergy, and exergoeconomic viewpoint. Bamisile et al. [25] have simulated a multifunctional cogeneration system including a gas turbine, a Rankin cycle with a two-pressure steam generator, an absorption chiller cycle, an ejector refrigeration cycle, a domestic hot water HX, and an electrolyzer. Loy-Benitez et al. [26] have investigated a combined cycle of gas turbines and organic Ranking cycle (ORC) for simultaneous production of heat and power from energy, exergy, and exergoeconomic perspective. Dhahad et al. [27] have studied a novel CCHP system in which a SOFC is coupled with ARC and ejector refrigeration cycles. An analysis of this system takes into account factors such as energy, efficiency, and economic efficiency. On the other hand, the critical technical and economic performance parameters of the system are examined, including the SOFC input temperature, ammonia concentration, and hot temperature difference of the generator.

In this century the cooling required for air conditioning is mainly provided by the vapor compression refrigeration cycle and the most important disadvantage of that is the dependence on high levels of electrical energy for operation. Recently, some cycles for cooling production have been introduced that use low or medium temperature thermal energy as a stimulus, which is called cold production cycles with thermal energy, and its types can be called absorption and compression chiller, ejector refrigeration cycle, combined Rankine cycle and heat pump. As mentioned, absorption chillers are one of the types of refrigeration production cycles with thermal energy stimulation, which have received a lot of attention today due to their ability to use clean and renewable energy at low temperatures. The common combinations in these systems are lithium LiBr–H2O and H_2_O–NH_3_, which in the first, water and the second ammonia are considered as refrigerants.

Anand Sinha et al. [28] examined the gas turbine exhaust waste heat generated for powering the fuel cell and made a comparative sustainable, qualitative and quantitative performance comparison between SGT, RGT and SOFC-RGT. Also in another study, gas turbine exhaust waste heat has been examined to power fuel cell and two typical intercooled gas turbines and a fuel-cell hybrid system were compared [29]. Kumar et al. [30] could obtain the reduction of power needed by air compressor using the intercooling heat to augment the extra outputted energy through organic Rankine cycle.

Among the researches conducted in the field of the absorption refrigeration cycle (ARC), Georgousis et al. [31] have performed energy and exergy analysis for a single stage LiBr–H_2_O absorption chiller considering the temperatures of hot water, cool water, and cold water entering the absorption chiller. Sotoodeh et al. [32] have done energy and exergy analysis for single stage and two-stage LiBr–H_2_O absorption chillers and calculated exergy destruction in each component of the absorption chiller. The highest amount of exergy destruction occurred in the absorber of the absorption chiller and then in the condenser, evaporator, generator, and HX, respectively. Nedaei et al. [33] have performed exergoeconomic analysis for a two-stage LiBr–H2O absorption chiller. They performed exergoeconomic analysis for a two-stage LiBr–H2O absorption chiller. Finally, the exergy flow of the fuel, product, and exergy destruction in the various components of the chiller were calculated to obtain the exergoeconomic factors in each component. The highest values of this factor were obtained for solution pump, generator, solution HX, and absorbent, condenser and, evaporator sets, respectively. Sleiti et al. [34] have performed energy and exergy analysis for a single-stage H_2_O–NH_3_ absorption chiller with a solar energy stimulant. Then they examined the effect of temperature change in different parts of the cycle on system performance in terms of energy and exergy perspective. The results showed that the highest irreversibility among the components of the absorption chiller occurs first in the absorber and then in the generator and condenser. Zare [35] has designed a hybrid system using a gas turbine with biomass fuel, in which waste heat is generated via exhaust gas is used to drive an H_2_O–NH_3_ ARC for cooling of compressor intake air. A practical, applied method is being developed and applied to improve open-cycle gas turbine power plants. Also, a thermoeconomic investigation and multi-objective optimization is carried out.

In the current investigation, a combined system including SOFC cycle, domestic hot water HX, and single stage H_2_O–NH_3_ absorption chiller are considered to simultaneously produce power, cold and hot water, and simulated from the energy, exergy, and exergoeconomic viewpoint is examined. The H_2_O–NH_3_ absorption cycle has so far received less attention than the LiBr–H2O absorption cycle, especially from the point of view of economic exergy, therefore the absorption cycle of H_2_O–NH_3_ is considered to produce refrigeration. Also, the study of exergoeconomics performance of SOFC, cogeneration cycle, hot water HX, and ARC are some of the innovations of this research that have not been done in other researched.

## 2 Description of the combined cycle performance

**Fig. 1:**
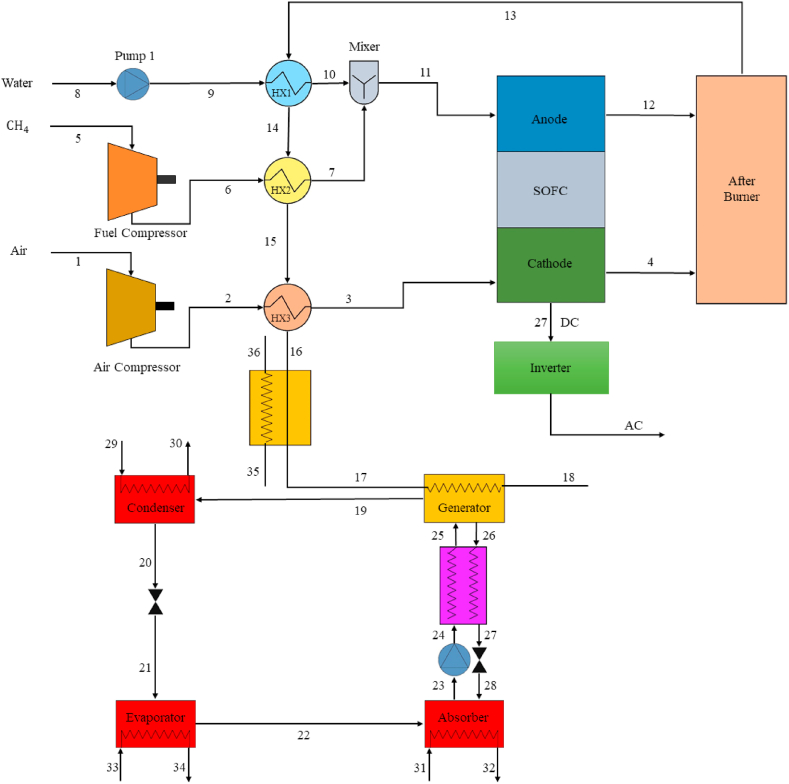
Schematic of a combined cycle.

Fig. 1 shows the flow diagram of the SOFC-Adsorption integration. Inlet air and fuel, and inlet water (at ambient temperature and pressure) are compressed to the operating pressure of the fuel cell by the compressor and pump respectively. In the next step, the increment of temperature in the three mentioned streams is done through the heat of the waste gas output from the afterburner by the air, fuel, and water HX, and the temperature and pressure of the three streams increase up to the temperature and pressure of the fuel cell inlet. High temperature compressed air enters the cathode of the fuel cell and as well, fuel and vapor enter the anode of the fuel cell then the output power of the fuel cell is achieved by the relevant reactions. In the next step, the excess and unreacted fuel with the anode reacts with the exhaust air from the cathode in the afterburner and the exhaust gas temperature increases. The temperature of hot gas produced is reduced in four stages. The waste gas temperature is reduced and hot water required for domestic use is produced through three air, fuel, and water HXs, and also in the hot water HX. Finally, in the H_2_O–NH_3_ ARC, the incoming waste gas acts as a stimulus for the generator.

The condenser and absorber of the absorption cycle are cooled with water at ambient temperature and the desired cold output is produced in the evaporator. As shown in Fig. 1, the absorption cycle consists of four main components: evaporator, absorber, generator, and condenser. In the evaporator, the liquid refrigerant evaporates at low pressure by absorbing heat from the external fluid and produces cold. then, in the absorber, the refrigerant vapor is absorbed into the sorbent and the solution is sent to the generator by a pump. External heat in the generator causes the temperature of the solution to rise and the vapor of the refrigerant separates from the solution. In the condenser, the refrigerant vapor produced in the generator is cooled and distilled by heat dissipation.

Then the liquid refrigerant produced in the condenser enters the evaporator by reducing the pressure through the solution valve, and the concentrated solution generated in the generator is sent to the absorber to reabsorb the vapor of refrigerant produced in the evaporator. Usually, an HX is located in the reciprocating path of the high temperature concentrated solution and the low temperature dilute solution between the generator and the absorber, which causes heat recovery and thus increases the efficiency of the absorption cycle. To simulate the hybrid cycle, the following simplifying assumptions are considered [[36], [37], [38]].

1. The system works in steady state.
2. Kinetic energy and potential changes in different components are ignored.
3. Air enters the fuel cell with a molar percentage of 79 nitrogen and 21% oxygen.
4. In a fuel cell, the fuel, water vapor, and air are combined at the same temperature.
5. The temperature of output products related to the anode and cathode as they leave the fuel cell is the same.
6. There is chemical equilibrium among the exhaust gases of the fuel cell.
7. Each component of the combined cycle has adiabatic function.
8. Ideal gas relations are used to determine the properties of mixtures of air, fuel, and gas.
9. The compressor and pump operate at constant isentropic efficiencies.
10. The fuel cell pressure drop is assumed to be 2% and also the afterburner pressure drop of 3%.
11. The pressure drop in the HXs of the above cycle has been assumed to be 2% however, the pressure drop in the HXs of the absorption cycle has been supposed to be zero.
12. The minimum temperature of exhaust gases is considered to be 101° Celsius to prevent the formation of corrosive substances.
13. Also, the H_2_O–NH_3_ solution leaving the generator and the absorber are saturated liquid.
14. For the analysis of exergy, ambient temperature and pressure are considered as referenced temperature and pressure.

## 3 Methodology

### 3.1 Combined cycle simulation

In this section, first, the general relationships related to energy and exergy analysis of components are described, and in section 3.1, the relationships related to SOFC discussed, which is a major component of the combined cycle, are described. To the thermodynamic analysis of the system, the equilibrium equations of energy, mass, and exergy for different parts of the system are written according to Eqs. (1), (2), (3), (4) [39].

(1) $$\sum \dot{m_{i}}=\sum \dot{m_{e}}$$

(2) $$\sum \dot{m_{i}}x_{i}=\sum \dot{m_{e}}x_{e}$$

(3) $$\dot{Q}+\sum \dot{m_{i}}h_{i}=\dot{W}+\sum \dot{m_{e}}h_{e}$$

(4) $$\dot{{Ex}_{Q}}+\dot{m_{i}}{ex}_{i}=\dot{m_{e}}{ex}_{e}+\dot{{Ex}_{W}}+\dot{{Ex}_{D}}$$

In which, i and e are related to the input and output of the control volume, respectively, and $\dot{{Ex}_{D}}$ is the exergy destruction in each component, and $\dot{{Ex}_{Q}}$ is the exergy corresponding to the heat transfer, $\dot{{Ex}_{W}}$ is the exergy corresponding to the power, and ex is the exergy corresponding to each flow which will be obtained from Eqs. (5), (6), (7) [40].

(5) $${\dot{Ex}}_{Q}=\left(1-\frac{T_{0}}{T_{k}}\right)\dot{Q_{i}}$$

(6) $${\dot{Ex}}_{W}=\dot{W}$$

(7) $$ex={ex}_{ph}+{ex}_{ch}$$

${ex}_{ph}$ and ${ex}_{ch}$ are physical and chemical exergies, respectively, defined as Eqs. (8), (9) [40].

(8) $${ex}_{ph}=h-h_{0}+T_{0}\left(s-s_{0}\right)$$

(9) $${ex}_{ch}=\left(\sum_{i=1}^{n}x_{0}{ex}_{i}^{ch}+RT_{0}\sum_{i=1}^{n}x_{i}\;\ln\;x_{i}\right)$$

Index 0 is related to the properties at ambient temperature and pressure and in Eq. (9), $x_{i}$ and ${ex}_{i}^{ch}$, are the standard molar fraction and chemical exergy of each component respectively. Also, to truly measure the efficiency of each component in the combined cycle, the exergy efficiency is defined according to Eq. (10) [41].

(10) $$\eta_{ex}=\frac{{\dot{Ex}}_{p}}{{\dot{Ex}}_{F}}=1-\frac{{\dot{Ex}}_{p}}{{\dot{Ex}}_{F}}$$

${\dot{Ex}}_{p}$ and ${\dot{Ex}}_{F}$ are related to the exergy flow of the product and the fuel of each component, respectively. The exergy flow of fuel represents the source that is the stimulator and ultimately produces the product, and the exergy flow of the product is the utilized exergy flow from a system that is according to the thermodynamic product of the system.

### 3.2 Solid oxide fuel cell (SOFC)

The reactions performed inside the fuel cell stack are reforming, shifting, and general electrochemical reaction which proceeds in Eq. (11), (12), (13)) at molar rates of $x_{r}$, $y_{r}$, and $z_{r}$, respectively [42].

(11) $$x_{r}\to \left(CH_{4}+H_{2}O\to CO+3H_{2}\right)$$

(12) $$y_{r}\to \left(CO+H_{2}O\to CO_{2}+H_{2}\right)$$

(13) $$z_{r}\to \left(H_{2}+\frac{1}{2}O_{2}\to H_{2}O\right)$$

In this research, because of high temperature of the operating SOFC and the less price, the reforming reaction inside the fuel cell is used, so that the reaction of reforming is performed inside the anode of the fuel cell and in this case, no external reforming is required. The needed heat of the reforming reaction is also utilized by the released heat from the other two reactions, namely the general electrochemical reaction and the shifting reaction. Assuming that the reforming reaction is done completely [9,37] (because of the high heat of the fuel cell and the catalytic effect of the materials used in the fuel cell anode), the shifting reaction of equilibrium constant can be calculated according to Eq. (14).

(14) $$\ln\left(K_{s}\right)=\frac{y_{r}.\left(3x_{r}+y_{r}-z_{r}\right)}{\left(x_{r}-y_{r}\right).\left(1.5x_{r}-y_{r}+z_{r}\right)}$$

However, calculation of the shifting reaction equilibrium constant is possible through Eq. (15) from the temperature of the exhaust gases of the fuel cell [43].

(15) $$\log\left(K_{s}\right)=A_{s}{\left(t_{SOFC,out}\right)}^{4}+B_{s}{\left(t_{SOFC,out}\right)}^{3}+C_{s}{\left(t_{SOFC,out}\right)}^{2}+D_{s}\left(t_{SOFC,out}\right)+Es$$

In which, the constants in Eq. (15) are given in Reference [43]. To obtain the molar rate of progress of the fuel cell triple reactions, in addition to the equilibrium constant equation of the shifting reaction, two equations of current density j and fuel utilization factor $U_{f}$ are also used [44].

(16) $$j=\frac{2F{.Z}_{r}}{N_{fc}.A_{a}}$$

(17) $$U_{f}=\frac{{\left(Fuel\right)}_{Consumed}}{{\left(Fuel\right)}_{Supplied}}=\frac{{\left(H_{2}\right)}_{Consumed}}{{\left(H_{2}\right)}_{Supplied}}=\frac{z_{r}}{3x_{r}+y_{r}}$$

It should be noted that the constants in Eqs. (16), (17) are presented in Table 5. Through solution of three equations mentioned simultaneously, the corresponding coefficients are obtained. Then, by knowing these coefficients and considering the triple reactions inside the fuel cell, the molar rates of different gases at the inlet and outlet of the fuel cell anode and cathode are calculated from Eq. (18), (19), (20), (21), (22), (23), (24), (25), (26), (27) [45].

(18) $${\dot{n}}_{CH_{4},11}=x_{r}$$

(19) $${\dot{n}}_{H_{2}O,11}=r_{sc}.x_{r}$$

(20) $${\dot{n}}_{H_{2},12}=3x_{r}+y_{r}-z_{r}$$

(21) $${\dot{n}}_{CO,12}=x_{r}-y_{r}$$

(22) $${\dot{n}}_{CO_{2},12}=y_{r}$$

(23) $${\dot{n}}_{H_{2}O,12}=1.5x_{r}-y_{r}+z_{r}$$

(24) $${\dot{n}}_{O_{2},3}=\frac{z_{r}}{2U_{0}}$$

(25) $${\dot{n}}_{N_{2},3}=\frac{79}{23}{\dot{n}}_{O_{2},3}$$

(26) $${\dot{n}}_{O_{2},4}={\dot{n}}_{O_{2},3}-\frac{z_{r}}{2}$$

(27) $${\dot{n}}_{N_{2},4}={\dot{n}}_{O_{2},3}$$

In Eq. (19) $r_{sc}$ is the ratio of steam to carbon and in Eq. (24) $U_{0}$ is the coefficient of air consumption. The air utilization factor, which calculates the amount of excess air that enters to the cathode, is the temperature control and fuel cell performance parameter, which is calculated from the conservation of energy equation in the fuel cell [46]. To calculate the output power of the fuel cell, first, the passing current I by Eq. (28) is obtained, and then the work resulting from Eq. (29) is calculated [47].

(28) $$I=j.A_{a}$$

(29) $${\dot{W}}_{SOFC}=N_{FC}.I{.V}_{c}$$

In which, $V_{c}$ is the voltage of the fuel cell obtained by Eq. (30).

(30) $$V_{c}{=V}_{n}-V_{loss}$$

In which, $V_{n}$ and $V_{loss}$ are the produced Nernst voltage and the voltage drop inside the fuel cell, respectively, and the voltage drop is calculated by Eq. (31) [37].

(31) $$V_{loss}{=V}_{ohm}+V_{act}+V_{conc}$$

The voltage drop of the fuel cell set is also the accumulation of three types of voltage drop; activation, ohmic, and concentration. The equations in Table 1 have been used to obtain the voltage drops mentioned. The output voltage of Nernst is obtained from Eq. (32) [37].

(32) $$V_{n}=-\frac{\Delta \bar{g{}^{\circ}}}{2F}+\frac{\bar{R}\left(t_{SOFC,out}\right)}{2F}.\mathrm{Ln}\left(\frac{a_{H_{2},12}.a_{O_{2},4}^{0.5}}{a_{H_{2},12}}\right)$$

In which, $\bar{g{}^{\circ}}$ is a standard Gibbs function based on mole and $a_{i,12}$ is obtained from Eq. (33).

(33) $$a_{i,12}=y_{i,12}\frac{P_{12}}{P_{ref}}$$

In which, y is the molar fraction of different flow, and $p_{ref}$ is the reference pressure of 101.3 kPa. In this section, using the presented equations, the fuel cell voltage is calculated, and by using Eq. (29), the output work of the fuel cell is calculated.

(34) $${\dot{m}}_{11}h_{11}+{\dot{m}}_{3}h_{3}={\dot{m}}_{12}h_{12}+{\dot{m}}_{4}h_{4}+{\dot{W}}_{SOFC}$$

**Table 1:** Electrochemical equations [37].

Title	Equation
$V_{ohm}=R_{c}+\rho_{c}.L_{c}+\rho_{a}.L_{a}+\rho_{e}.L_{e}+\rho_{\mathrm{int}}.L_{\mathrm{int}}$	Ohmic voltage drop
$\rho_{c}={\left(\left(\frac{42E6}{t_{SOFC.out}}\right)*\exp\left(\frac{-1200}{t_{SOFC.out}}\right)\right)}^{-1}$	
$\rho_{a}={\left(\left(\frac{95E6}{t_{SOFC.out}}\right)*\exp\left(\frac{-1150}{t_{SOFC.out}}\right)\right)}^{-1}$	
$\rho_{c}={\left(\left(\frac{9.3E6}{t_{SOFC.out}}\right)*\exp\left(\frac{-1100}{t_{SOFC.out}}\right)\right)}^{-1}$	
$V_{act}=V_{act,a}+V_{act,c}$	Activation voltage drop
$V_{act,a}=\frac{\bar{R}.\left(t_{SOFC.out}\right)}{F}.\sinh^{-1}\left(\frac{j}{2j_{oa}}\right)$	
$V_{act,c}=\frac{\bar{R}.\left(t_{SOFC.out}\right)}{F}.\sinh^{-1}\left(\frac{j}{2j_{oc}}\right)$	
$V_{Conc}=V_{Conc,a}+V_{Conc,c}$	Concentration voltage drop
$V_{Conc,a}=\frac{\bar{R}.\left(t_{SOFC.out}\right)}{2F}\left(\mathrm{Ln}\left(1+\frac{P_{H_{2},12}.j}{P_{H_{2},12}.j_{as}}\right)-\mathrm{Ln}\left(1-\frac{j}{j_{as}}\right)\right)$	
$V_{Conc,c}=-\left(\frac{\bar{R}.\left(t_{SOFC.out}\right)}{2F}\right).\mathrm{Ln}\left(1-\frac{j}{j_{cs}}\right)$	
$j_{as}=\frac{2F{.P}_{H_{2},12}.D_{aeff}}{\bar{R}.\left(t_{SOFC.out}\right).L_{a}}$	
$j_{cs}=\frac{4F{.P}_{O_{2},4}.D_{ceff}}{\left(\frac{P_{4}-P_{O_{2},4}}{P_{4}}\right)\bar{R}.\left(t_{SOFC.out}\right).L_{c}}$	

The last equation of the fuel cell set equations could be the energy conservation and due to the adiabatic nature of the fuel cell, it can be written as Eq. (34).

Finally, the output power of the fuel cell is converted to direct electric current by the inverter as shown in Eq (35).

(35) $${\dot{W}}_{SOFC,DC}={\dot{W}}_{SOFC}\eta_{inverter}$$

### 3.3 Exergoeconomical analysis

The exergy costing process consists of cost equilibrium equations are mentioned separately for each component of system according to Eq. (36).

(36) $$\sum {\left(c_{e}{\dot{Ex}}_{e}\right)}_{k}+c_{w,k}{\dot{W}}_{k}=c_{Q,k}{\dot{Ex}}_{Q,k}+\sum {\left(c_{i}{\dot{Ex}}_{i}\right)}_{k}+{\dot{Z}}_{k}$$

In which, c is the unit cost of exergy, ${\dot{Z}}_{k}$ is the cost rate for the k component and it is obtained from Eq. (37).

(37) $${\dot{Z}}_{k}=\frac{Z_{k}.CRF.\varphi }{N}$$

In which, $Z_{k}$ is the initial cost of purchasing of the component (presented in Table 2 [10,48] updated using the 2013 price-related relationships), φ is the cost of operating and maintaining of the component, N is the number of annual operating hours of the component, and CRF is the capital recovery factor obtained from Eq. (38).

(38) $$CRF=\frac{i{\left(1+i\right)}^{n}}{{\left(1+i\right)}^{n}-1}$$

In which i is the interest rate equal to 10%, N is the number of years of system operation equal to 20 years. Also, φ=1.06 and N=7446 hours are considered [10]. The economic equations in different components, together with the auxiliary equations using the SPECO method, create a set of linear equations, by solving them the unit cost of exergy of all floware calculated [49]. The main and auxiliary equations for the exergoeconomic analysis of the various components of the combined fuel cell cycle and the triple recovery cycle are presented in Table 2.

**Table 2:** The cost function of different components, cost balance, and auxiliary equations for exergoeconomic analysis of the combined cycle [10,48].

Component	Initial price	Main relation	Auxiliary relation
Air compressor	${\dot{Z}}_{air,com}=71.1{\dot{m}}_{air}\left(\frac{1}{0.9-\eta_{com}}\right)\left(\frac{p_{2}}{p_{1}}\right)\ln\left(\frac{p_{2}}{p_{1}}\right)$	$\dot{C_{1}}+c_{el,sofc}{\dot{W}}_{air,com}+{\dot{Z}}_{air,com}={\dot{C}}_{2}$	$c_{1}=0$
Fuel compressor	${\dot{Z}}_{fuel,com}=71.1{\dot{m}}_{fuel}\left(\frac{1}{0.9-\eta_{com}}\right)\left(\frac{p_{6}}{p_{5}}\right)\ln\left(\frac{p_{6}}{p_{5}}\right)$	$\dot{C_{5}+c_{el,sofc}{\dot{W}}_{air,com}+{\dot{Z}}_{air,com}={\dot{C}}_{6}}$	cfuel=12($Gj)
Water pump	${\dot{Z}}_{water,p}=705.48{{\dot{W}}_{water,p}}^{0.71}\left(1+\frac{0.2}{1-\eta_{water,p}}\right)$	${\dot{C}}_{8}+c_{el,sofc}{\dot{W}}_{air,com}+{\dot{Z}}_{air,com}={\dot{C}}_{9}$	$c_{8}=0$
Air HX	${\dot{Z}}_{air,hx}=3.130{\left(\frac{A_{air,hx}}{0.093}\right)}^{0.78}$	${\dot{C}}_{2}+{\dot{C}}_{15}+{\dot{Z}}_{air,hx}={\dot{C}}_{3}+{\dot{C}}_{16}$	$c_{15}=c_{15}$
Fuel HX	${\dot{Z}}_{fuel,hx}=130{\left(\frac{A_{fuel,hx}}{0.093}\right)}^{0.78}$	${\dot{C}}_{6}+{\dot{C}}_{14}+{\dot{Z}}_{fuel,hx}={\dot{C}}_{7}+{\dot{C}}_{15}$	$c_{14}=c_{15}$
Water HX	${\dot{Z}}_{water,hx}=130{\left(\frac{A_{water,hx}}{0.093}\right)}^{0.78}$	${\dot{C}}_{9}+{\dot{C}}_{13}+{\dot{Z}}_{water,hx}={\dot{C}}_{10}+{\dot{C}}_{14}$	$c_{13}=c_{14}$
Fuel cell	$Z_{SOFC}=A_{a}N_{fc}\left(2.96t_{SOFC,out}-1907\right)$	${\dot{C}}_{3}+{\dot{C}}_{11}+{\dot{Z}}_{SOFC}={\dot{C}}_{12}+{\dot{C}}_{4}+c_{26}{\dot{W}}_{SOFC}$	$c_{4}=c_{26}$ $c_{12}=c_{26}$
Inverter	$Z_{inverter}=100000{\left(\frac{{\dot{W}}_{SOFC}}{500}\right)}^{0.7}$	$c_{26}{\dot{W}}_{SOFC}+{\dot{Z}}_{of,eva}=c_{el,SOFC}{\dot{W}}_{SOFC,ac}$	−
Afterburner chamber	$Z_{AB}=46.08{\dot{m}}_{4}\left(\frac{1+\exp\left(0.018t_{13}-26.4\right)}{0.995-\frac{p_{13}}{p_{12}}}\right)$	${\dot{C}}_{4}+{\dot{C}}_{12}+{\dot{Z}}_{AB}={\dot{C}}_{13}$	−
Hot water HX	$Z_{of,hx}=130{\left(\frac{A_{of,hx}}{0.093}\right)}^{0.78}$	$c_{16}{\dot{Ex}}_{16}+c_{35}{\dot{Ex}}_{35}+{\dot{Z}}_{dhw,hx}=c_{17}{\dot{Ex}}_{17}+c_{36}{\dot{Ex}}_{36}$	$c_{16}=c_{17}$
Generator	$Z_{gen,ACH}=0.322\left(30000+0.75{A_{gen}}^{0.8}\right)$	$c_{17}{\dot{Ex}}_{17}+c_{25}{\dot{Ex}}_{25}+{\dot{Z}}_{gen}=c_{18}{\dot{Ex}}_{18}+c_{19}{\dot{Ex}}_{19}+c_{26}{\dot{Ex}}_{26}$	$\frac{c_{19}{\dot{Ex}}_{19}-c_{25}{\dot{Ex}}_{25}}{{\dot{Ex}}_{19}-{\dot{Ex}}_{25}}=\frac{c_{25}{\dot{Ex}}_{25}-c_{26}{\dot{Ex}}_{26}}{{\dot{Ex}}_{26}-{\dot{Ex}}_{25}}$
Absorber	$Z_{abs,ACH}=0.322\left(30000+0.75{A_{abc}}^{0.8}\right)$	$c_{22}{\dot{Ex}}_{22}+c_{28}{\dot{Ex}}_{28}+c_{31}{\dot{Ex}}_{31}+{\dot{Z}}_{abs}=c_{23}{\dot{Ex}}_{23}+c_{32}{\dot{Ex}}_{32}$	$c_{22}=c_{23}$ $c_{31}=0$
Condenser	$Z_{con,ACH}=0.322\left(30000+0.75{A_{con}}^{0.8}\right)$	$c_{19}{\dot{Ex}}_{19}+c_{29}{\dot{Ex}}_{29}+{\dot{Z}}_{con}=c_{20}{\dot{Ex}}_{20}+c_{30}{\dot{Ex}}_{30}$	$c_{19}=c_{20}$ $c_{29}=0$
Evaporator	$Z_{eva,ACH}=0.322\left(30000+0.75{A_{eva}}^{0.8}\right)$	$c_{21}{\dot{Ex}}_{21}+c_{33}{\dot{Ex}}_{33}+{\dot{Z}}_{eva}=c_{22}{\dot{Ex}}_{22}+c_{34}{\dot{Ex}}_{34}$	$c_{21}=c_{22}$ $c_{33}=0$
Solution HX	$Z_{shx,ACH}=0.322\left(30000+0.75{A_{shx}}^{0.8}\right)$	$c_{24}{\dot{Ex}}_{24}+c_{26}{\dot{Ex}}_{26}+{\dot{Z}}_{shx}=c_{23}{\dot{Ex}}_{23}+c_{32}{\dot{Ex}}_{32}$	$c_{26}=c_{27}$
Solution pump	$Z_{p,ACH}=1120{{\dot{W}}_{p,ACH}}^{0.8}$	$c_{23}{\dot{Ex}}_{23}+c_{el}{\dot{W}}_{sp}+{\dot{Z}}_{sp}=c_{24}{\dot{Ex}}_{24}$	cel=10($Gj)

The mean cost per unit of fuel and product and the cost of exergy destruction for the k_th_ component of the system are calculated by Eq. (39)–(41).

(39) $$c_{F,k}=\frac{{\dot{C}}_{F,k}}{{\dot{Ex}}_{F,k}}$$

(40) $$c_{P,k}=\frac{{\dot{C}}_{P,k}}{{\dot{Ex}}_{P,k}}$$

(41) $${\dot{C}}_{dest,k}=c_{F,k}{\dot{Ex}}_{D,k}$$

Finally, exergoeconomic factor $f_{k}$ is obtained according to Eq. (42) [40].

(42) $$f_{k}=\frac{{\dot{Z}}_{k}}{{\dot{Z}}_{k}+{\dot{C}}_{dest,k}}$$

### 3.4 Output parameters

The fit power outputted, efficiency of exergy analysis, and complete rate of cost of the hybrid cycle are computed using Eq. (43), (44), (45)) to examine the combined cycle performance.

(43) $${\dot{W}}_{net}={\dot{W}}_{SOFC,DC}-{\dot{W}}_{air,com}-{\dot{W}}_{fuel,com}-{\dot{W}}_{water,p}$$

(44) $$\eta_{ex}=\frac{{\dot{W}}_{net}+\left({\dot{Ex}}_{36}-{\dot{Ex}}_{35}\right)+\left({\dot{Ex}}_{34}-{\dot{Ex}}_{33}\right)}{{\dot{Ex}}_{5}}$$

(45) $${\dot{C}}_{tot}=\sum {\dot{Z}}_{k}+\sum {\dot{C}}_{Dest,k}+{\dot{C}}_{fuel}$$

That, fuel cost rate is calculated from Eq. (46).

(46) $${\dot{C}}_{fuel}={\dot{m}}_{fuel}c_{fuel}\left({LHV}_{fuel}\right)$$

## 4 Result and discussion

### 4.1 Basic mode results

All mass and energy conservation equations and irreversibility equations, as well as equations related to exergoeconomic analysis of the combined cycle's various components, are simulated by EES software. Initially, to validate the results, the output results of the SOFC, with the same input cases, were compared with reference [37] in Table 3. As can be seen, there is a good correspondence between the results obtained and the results of the mentioned reference. Initial values for simulation are shown in Table 4.

**Table 3:** Validation of the present simulation related to SOFC.

Parameter	Present study	Reference [37]
$V_{n}\left(v\right)$	0.8431	0.843
$V_{loss}\left(v\right)$	0.2887	0.2881
$W_{SOFC}\left(kW\right)$	478.9	489.211

**Table 4:** Initial values for simulation [36,37].

Parameter	Description	Value
$t_{0}\left({}^{\circ}C\right)$	Ambient temperature	25
$p_{0}\left(kPa\right)$	Ambient pressure	101.3
$r_{p,com}$, $r_{p,pump}$	Compressor and pump pressure ratio	1.19
$\eta_{com}$	Isentropic efficiency of air and fuel compressors	0.85
$\eta_{water,p}$	Isentropic efficiency of water pump	0.85
$\eta_{AB}$	After burner chamber efficiency	0.99
$t_{35}\left({}^{\circ}C\right)$	Inlet water temperature of hot water HX	25
$p_{33}\left(kPa\right)$	Inlet water pressure of hot water HX	101.3
$t_{36}\left({}^{\circ}C\right)$	Outlet water temperature of hot water HX	25
$t_{19}\left({}^{\circ}C\right),t_{26}\left({}^{\circ}C\right)$	Absorption chiller generator temperature	80
$t_{23}\left({}^{\circ}C\right)$	Absorption temperature	30
$t_{20}\left({}^{\circ}C\right)$	Condenser temperature	30
$t_{22}\left({}^{\circ}C\right)$	Evaporator temperature	2
$\Delta T_{gas}\left({}^{\circ}C\right)$	Inlet and outlet exhaust gas temperature difference of the generator	8
$t_{29}\left({}^{\circ}C\right),t_{31}\left({}^{\circ}C\right)$	The temperature of the inlet cold water of condenser and absorber	25
$p_{29}\left(kPa\right)$	The inlet pressure of the cool water of condenser and absorber	101.3
$t_{32}\left({}^{\circ}C\right),t_{30}\left({}^{\circ}C\right)$	The temperature of the outlet cold water of condenser and absorber	35
$t_{33}\left({}^{\circ}C\right)$	Inlet cold water temperature of the evaporator	12
$p_{33}\left(kPa\right)$	Inlet cold water pressure of the evaporator	101.3
$t_{34}\left({}^{\circ}C\right)$	Outlet cold water temperature of the evaporator	7
$\varepsilon_{shx}$	Efficiency of solution HX	0.8
$\eta_{sp}$	Efficiency of solution pump	0.8
F(Cmol)	Faraday constant	96485
$t_{SOFC,in}\left({}^{\circ}C\right)$	Fuel cell inlet temperature	726.85
$t_{SOFC,out}\left({}^{\circ}C\right)$	Fuel cell outlet temperature	826.85
$U_{f}$	Fuel utilization factor of fuel cell	0.85
$A_{a}\left(m^{2}\right)$	Area of single fuel cell	0.01
j(Am2)	Current density	8000
$\eta_{inverter}$	Inverter efficiency	0.97
jas(Am2)	Anode current density	6500
jcs(Am2)	Current density of cathode	2450
Daeff(m2s)	Cathode effective diffusion coefficient	2E-5
Dceff(m2s)	Anode effective diffusion coefficient	5E-6
$N_{fc}$	Number of fuel cells	11000
$r_{sc}$	Vapor to carbon ratio	2.5
$L_{a}\left(m\right)$	Anode thickness	5E-4
$L_{c}\left(m\right)$	Cathode thickness	2E-5
$L_{e}\left(m\right)$	Electrolyte thickness	1E-5
$L_{\mathrm{int}}\left(m\right)$	Connection thickness	0.003

To simulate the combined cycle, the initial inputs of the cycle in the base state are as shown in Table 5 [36,37]. Using the input values and also mass and energy balance equations and the equations related to exergy analysis and exergoeconomic; values of output related to the rate of energy, rate of exergy, exergoeconomics, and exergy efficiency of the various components of the hybrid system are presented in Table 5. As can be seen, the greatest amount of exergy destruction occurs in air HX, water HX, and fuel cells. Also, the highest amount of exergy efficiency is in compressors and solution valves and the lowest amount of that is in the hot water HX and absorption chiller. Also in the input base state, the total power is 418 kW, the total irreversibility is 650.1 kW and the total exergy efficiency is 0.378.

**Table 5:** Output result of the energy rate, different exergies, exergoeconomic and exergy efficiency of the components of combined cycle.

Component	$\dot{Q}or\dot{W}\left(kW\right)$	${\dot{Ex}}_{f}\left(kW\right)$	${\dot{Ex}}_{p}\left(kW\right)$	${\dot{Ex}}_{D}\left(kW\right)$	$\eta_{ex}$	cF=($Gj)	cP=($Gj)	C˙D=($h)	Z˙=($h)	f(%)
Air compressor	54.05	54.05	46.37	7.679	0.857	29.665	34.693	0.82	0.018	2.147
Fuel compressor	0.717	0.717	0.614	0.103	0.856	29.665	34.693	0.011	0.00013	1.199
Water pump	0.0014	0.0014	0.0012	0.0002	0.817	29.665	107.135	0.000028	0.0003	91.43
Air HX	2206	1316	1108	208.2	0.841	29.665	36.248	22.1	4.958	18.33
Fuel HX	52.2	43.4	27.49	15.91	0.633	29.664	46.665	1.689	0.038	2.219
Water HX	242.57	203.2	75.87	127.3	0.373	29.664	79.247	13.51	0.087	0.6417
Fuel cell	473.3	598.6	487.9	110.6	0.815	21.254	27.702	8.446	2.858	25.24
Afterburner	–	1808	1701	107.8	0.94	27.702	29.664	10.75	0.111	1.029
Hot water HX	246.1	76.49	13.52	62.97	0.176	29.664	174.134	6.682	0.359	–
Generator	25.86	10.92	3.18	7.744	0.291	29.644	118.217	0.821	0.183	18.26
Absorber	23.74	0.998	0.401	0.597	0.401	187.6	594.15	0.403	0.183	31.26
Condenser	17.35	0.429	0.293	0.136	0.683	141.079	380.544	0.069	0.183	72.65
Evaporator	15.15	1.257	0.823	0.434	0.654	141.134	277.492	0.22	0.183	45.41
Solution HX	10.3	1.08	0.677	0.402	0.627	141.134	300.269	0.204	0.183	47.28
Solution pump	0.077	0.077	0.061	0.015	0.795	10	23.571	0.0005	0.0024	81.06
Solution pressure relief valve	–	516.4	516.3	0.045	0.999	141.134	141.218	0.022	–	–
Refrigerant pressure relief valve	–	271.1	271	0.108	0.999	141.079	141.107	0.055	–	–

In this research, air HX, hot water HX, and fuel cell are mentioned as parts that should be examined more than other parts from exergoeconomic viewpoint since almost the most significant amount of ${\dot{Z}}_{k}+{\dot{C}}_{D,k}$ belongs to these components. Also, as shown in Table 5, the fuel compressor, after burner, and water HX have the lowest amount of factor of exergoeconomic. The obtained numbers for the mentioned parts mean that the cost of destruction of exergy is greater than the primarily cost, and as the result of that the factor of exergoeconomic of each part is reduced. In the case of these parts in the combined system, an increase in the primarily price is suggested to decrease the amount of destruction for exergy. That is achieved in the HX by enhancing the area of the exchanger and on the other hand by reducing the exchanger temperature difference (which leads to a reduction in irreversibility in the exchanger). Also, the unit price of electrical generated power by the inverter is 29,665 dollars per Gj. Finally, it should be noted that the total value of factor of exergoeconomic for the combined system is 12.44%. This amount shows that 87.56% of the cost of the cycle is because of the cost related to the destruction of exergy. It is concluded that by using parts with above prices that decrease the cost of exergy and increase the initial price of the system, the performance of the system from the perspective of exergoeconomic will be improved.

### 4.2 Results of parametric analysis

**Fig. 2:**
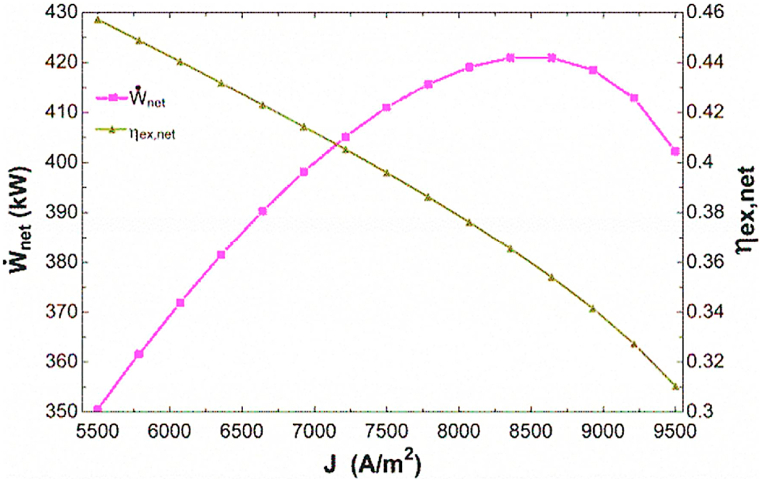
The impact of changing in current density on total power and exergy efficiency.

**Fig. 3:**
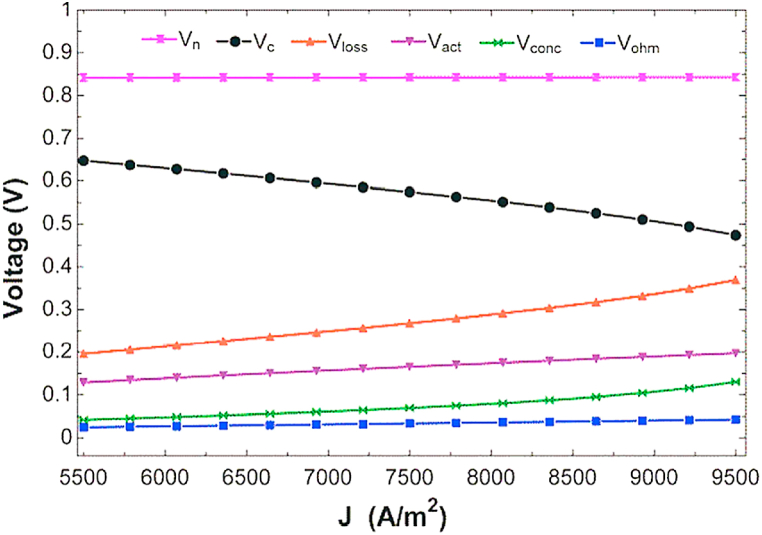
The impact of changing in current density on different voltage.

In this section, the effect of changes in fuel cell current density and fuel utilization factor on system performance from the perspective of energy, exergy, and economic exergy has been investigated. It should be noted that in order to parametric analysis, the parameters of only the desired ones are altered in the considered interval and the rest of the input items in the combined cycle continue to be constant in the base state according to Table 5. Fig. 2 shows the effect of changing the fuel cell passing current density on the work and overall exergy efficiency. As can be seen in Fig. 2, by increasing the current density in the desired range, the work has a maximum value, and the efficiency decreases. Increasing the passing current density through the fuel cell leads to increases the passing current and decreases the voltage of the fuel cell ($V_{c}$ in Fig. 3), which according to Eq. (29) leads to maximizing the output power of the fuel cell. Also the increment in the current density, leads to increase the flow rate of air, fuel and water required for the inlet, which increases the work required for the air, fuel compressor and water pump. Finally, increasing the output power of the fuel cell will increase the overall power output of cycle. Beside, increment in the current density leads to increase the flow rate of hot and cool water produced and the flow rate of methane fuel input. Despite increasing in the flow rate of hot and cold water produced in numerator of fraction of exergy efficiency, the effect of increasing the required methane flow rate at the denominator of exergy efficiency is greater and reduces the exergy efficiency.

**Fig. 4:**
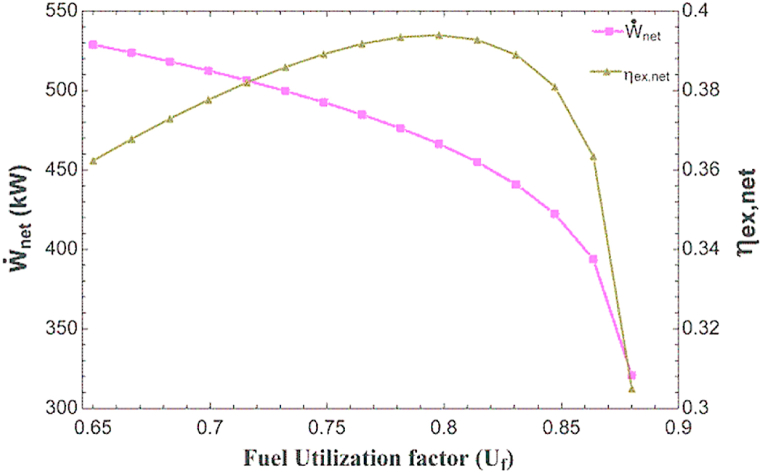
The impact of changing in fuel utilization factor on power and total exergy.

Fig. 4 shows the impact of changing the fuel cell fuel utilization factor on the work and overall exergy efficiency. As can be seen in this figure, by increasing the fuel utilization factor in the mentioned efficiency, the output energy is reduced and the efficiency of exergy reaches to the maximum value. Increasing the fuel utilization factor reduces the voltage passing through the fuel cell, which, while remaining constant the rest cases, is the only effective factor in reducing the output power of the fuel cell. Also, increasing in the fuel utilization factor, increases the air flow rate and decrease the inlet fuel and water flow rate, which increases the work required by the air compressor and reduces the work required by the fuel compressor and water pump. Eventually, the overall power decreases in a manner similar to the decrement of the fuel cell power. Also, increment in the fuel utilization factor in addition to the mentioned cases, decreases the hot water flow rate produced and increases the flow rate of cold water produced, which ultimately leads to maximizing the exergy efficiency.

**Fig. 5:**
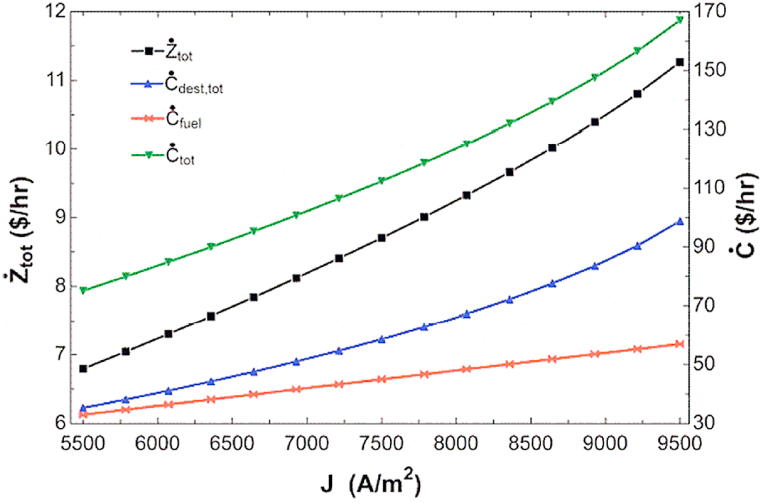
The impact of changing in current density on the cost rates.

Fig. 5 shows the impact of current density changing on initial cost rate, exergy destruction cost rate, fuel cost rate and total cost rate. Increasing in the current density of the fuel cell results in increasing the flow rate of different parts of the combined cycle, which is an effective factor in increasing the initial price of all components of the combined cycle. Among the components, only the initial price of the fuel cell remains unchanged. This increase in flow rate also increases the irreversibility and the cost rate of the irreversibility of the components in the combined cycle, and ultimately the total cost rate increases due to the increase of all its sentences.

**Fig. 6:**
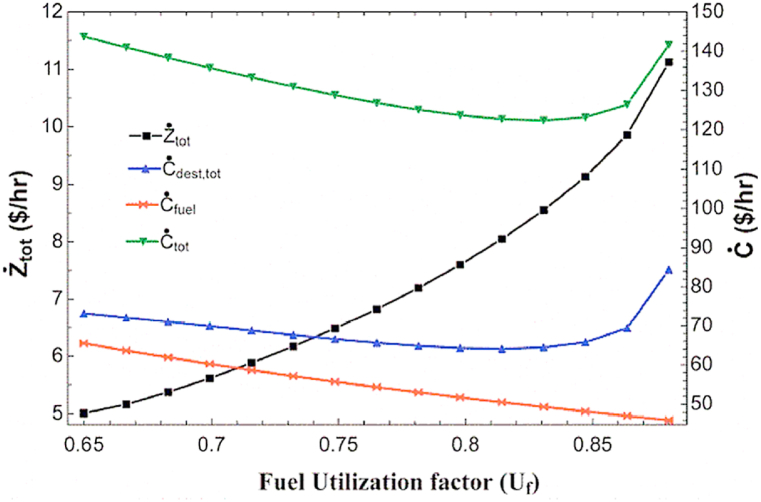
The impact of fuel utilization factor on different cost rate.

Fig. 6 shows the impact of changing the fuel cell fuel utilization factor on the initial cost rate, exergy destruction cost rate, fuel cost rate and total cost rate. Increasing the fuel utilization factor, as mentioned before, increases the required air flow rate and decreases the fuel and water flow rate required for the fuel cell inlet, as well as reduces the produced hot water flow rate and increases the produced cold water flow rate. Regarding the initial price rate, the mentioned changes in flow rate have a complex and contradictory effect on different components, which ultimately leads to an increase in the total initial price. However, in the case of exergy destruction cost rate, this cost is increased in all components of the combined cycle and decreases significantly in the hot water HX, which results the minimum exergy destruction cost rate. The total cost rate has a similar trend to the exergy destruction cost rate and has a minimum value in the fuel utilization factor of about 0.83.

## 5 Conclusion

In the present study, the combined production system of triple SOFC, HX for hot water production and H_2_O–NH_3_ ARC was studied and analyzed from the view of exergy, energy and exergoeconomic. The main achieved conclusions are as follows.

• The base state results showed the total power of 418.4 kW, the total irreversibility of 650.1 kW and the total exergy efficiency of 37.8% for the combined cycle system.
• The total amount of exergoeconomic factor for the combined cycle was 12.44%. This value indicated that 87.56% of the cost of system was due to the cost corresponding to the exergy destruction, and as a consequence, the use of higher priced elements improves the system performance in terms of exergoeconomics.
• The results of parametric analysis showed that increasing the current density reduces the exergy efficiency, creates the maximum for the overall power and increases all the cost rates. Increasing the fuel utilization factor also reduces the overall power, creating the maximum for the exergy efficiency and creating the minimum for the total cost rate.

## Author contribution statement

Rahim Zahedi: contributed reagents, materials, analysis tools or data.

Mohammad Mahdi Forootan: Wrote the paper; performed the experiments.

Mansour Keshavarzzadeh: analyzed and interpreted the data; conceived and designed the experiments.

Rouhollah Ahmadi: contributed reagents, materials, analysis tools or data.

## Data availability statement

Data will be made available on request.

## Declaration of competing interest

The authors declare that they have no known competing financial interests or personal relationships that could have appeared to influence the work reported in this paper.
